# Widespread *in situ* follicular neoplasia in patients who subsequently developed follicular lymphoma

**DOI:** 10.1002/path.5861

**Published:** 2022-03-03

**Authors:** Rachel Dobson, Andrew Wotherspoon, Shizhang Alexander Liu, Francesco Cucco, Zi Chen, Yuan Tang, Ming‐Qing Du

**Affiliations:** ^1^ Division of Cellular and Molecular Pathology, Department of Pathology University of Cambridge Cambridge UK; ^2^ Histopathology Department The Royal Marsden Hospital London UK; ^3^ Department of Pathology, West China Hospital Sichuan University Chengdu PR China; ^4^ Department of Histopathology, Addenbrooke’s Hospital Cambridge University Hospitals NHS Foundation Trust Cambridge UK

**Keywords:** *in situ* follicular neoplasia, follicular lymphoma, clonal evolution, somatic mutation

## Abstract

*In situ* follicular neoplasia (ISFN) is usually an occasional incidental finding in lymph nodes by BCL2 immunohistochemistry, and its true scale is unknown. We have identified six cases of follicular lymphoma (FL) with a history of solid neoplasm 4–16 years ago, from which ISFN was identified widely in the surgically cleared lymph nodes (LNs). Using clone‐specific PCR and BaseScope *in situ* hybridisation with primers or probes specific to the *VDJ* or *BCL2–IGHJ* junction sequence, we confirmed the clonal identity among different ISFNs and overt‐FL in each of the four cases successfully investigated. Mutation analyses of overt‐FL by targeted next‐generation sequencing identified multiple potential pathogenic changes involving *CREBBP*, *EZH2*, *KMT2D*, *TNFRS14*, and *STAT6*. Further investigations of these mutations in paired ISFNs using Fluidigm PCR and Illumina sequencing showed the presence of the FL‐associated mutations in early lesions for two of the six cases investigated (*CREBBP* and *KMT2D* in one case and *STAT6* in the other), with one case displaying stepwise accumulation of its observed mutations. Remarkably, there were considerable divergences in *BCL2* variants among different ISFN‐involved lymph nodes in all four cases successfully investigated, indicating ongoing intraclonal diversification by somatic hypermutation machinery. Our findings demonstrate widespread distribution of ISFN lesions, further implicating their dynamic nature with the neoplastic cells undergoing active trafficking and clonal evolution. © 2021 The Authors. *The Journal of Pathology* published by John Wiley & Sons Ltd on behalf of The Pathological Society of Great Britain and Ireland.

## Introduction

Follicular lymphoma (FL) is characterised by t(14;18)(q32;q21)/*IGH::BCL2* and expansion of the transformed translocation‐positive cells in B‐cell follicles. The translocation juxtaposes the *BCL2* gene to the immunoglobulin heavy chain joining region (*IGHJ*) and causes *BCL2* overexpression. The translocation is caused by erroneous genomic rearrangements during VDJ recombination at the pre‐B stage of B‐cell development in the bone marrow. Most breakpoints in the *BCL2* genes are clustered, thus allowing detection of the *BCL2–IGHJ* fusion by PCR [1].

Apart from lymphoma, the translocation is found in peripheral blood lymphocytes in more than 50% of healthy adults by PCR [2]. These circulating translocation‐positive cells are most likely equivalent to those of *in situ* follicular neoplasia (ISFN) in lymph nodes (LNs) identified incidentally by BCL2 immunohistochemistry [3, 4]. The B‐cell follicles involved by ISFN usually display only subtle changes that are not readily identifiable using conventional H&E staining but are recognised by strong BCL2 staining in germinal centre B‐cells [5]. Generally, few ISFNs progress into an overt B‐cell lymphoma. However, its true incidence remains unclear [3, 6].

There are limited studies documenting the evolution of ISFN to subsequent FL or other B‐cell lymphomas, and their underlying genetic changes [7, 8, 9]. Several FL‐associated somatic genetic changes have been found previously in ISFN, including *CREBBP*, *KMT2D*, *EZH2*, and *TNFRSF14* mutations [7, 8, 9]. However, most previous studies involve analysis of a single ISFN tissue specimen. Therefore, the true extent of ISFN lesions and their intraclonal genetic changes are unknown. We have identified six cases of FL with a history of solid neoplasia, from which ISFN was retrospectively identified widely in the surgically removed LNs. The present study reports the extent of ISFN lesions, their clonal relationship, and underlying genetic changes.

## Materials and methods

### Case and tissue materials

Local ethical guidelines were followed for the use of archival tissues for research with the approval of the ethics committee (05‐Q1604‐10). The study included six FL cases with a history of solid neoplasia 4–16 years ago, from which ISFN was identified widely in the surgically removed LNs by BCL2 immunohistochemistry (Table 1).

**Table 1:** Case information and number of LNs showing prominent ISFN involvement with at least one follicle displaying clustered or diffuse BCL2 positivity.

Case	Original diagnosis	Original therapy applied	ISFN in cleared lymph nodes	Follicular lymphoma (FL)	FL treatment
**A**	52 years/female: Left breast ductal carcinoma	Chemotherapy, followed by mastectomy and axillary lymph node clearance	Seen in 4/17 lymph nodes cleared	**6 years later** Right axillary lymph node biopsy, FL, grade 2, stage IV	Transformation to DLBCL 5 years after FL diagnosis, treated with R‐PMitCEBO, then R‐CODOX M‐IVAC, radiotherapy, R‐Bendamustine, died 17 years after FL diagnosis
**B**	45 years/female: Right breast ductal carcinoma	Lumpectomy and axillary lymph node clearance, followed by chemotherapy	Seen in 3/21 lymph nodes cleared	**16 years later** Left groin lymph node biopsy, FL, grade 1–2, stage III	Watch and wait for 2 years, then treated with R‐Bendamustine and R maintenance for 2 years, alive and well 8 years after FL diagnosis
**C**	48 years/female: Left lower leg melanoma	Surgical excision and ilio‐inguinal lymph node clearance	Seen in 14/19 lymph nodes cleared	**6 years later** Axillary lymph node biopsy, FL, grade 1, stage IIIa	R‐CVP ×6 and R maintenance for 2 years, relapsed 2 years later, treated with radiotherapy, then R‐Bendamustine in CONTRALTO trial (R‐Benda ± GDC‐0199), complete remission, alive and well 10 years after FL diagnosis
**D**	57 years/male: Gastric adenocarcinoma	Chemotherapy, followed by total gastrectomy	Seen in 4/30 lymph nodes cleared	**4 years later** Left level II neck lymph node, FL, grade 1, stage I	Radiotherapy (30 Gy), alive and well 12 years after FL diagnosis
**E**	49 years/male Metastatic moderately differentiated squamous cell carcinoma in a neck lymph node	Radiotherapy (50 Gy)	Seen in 3/4 lymph nodes cleared	**4 years later** Right neck lymph node, FL, grade 3a, stage I	Not available
**F**	48 years/male Pleomorphic salivary gland adenoma	Surgical excision	Seen in 1/2 lymph nodes cleared	**7.5 years later** tonsil, FL, grade 1–2, stage III	Watch and wait, alive and well 8 years after FL diagnosis

DLBCL, diffuse large B‐cell lymphoma; R, rituximab; PMitCEBO, prednisolone, mitoxantrone, cyclophosphamide, etoposide, bleomycin, and vincristine; CODOX M‐IVAC, cyclophosphamide, vincristine, doxorubicin, methotrexate, ifosfamide, etoposide, and cytarabine; CVP, cyclophosphamide, vincristine, and prednisone; Gy, Gray.

### Methodology outline

Detailed methods are provided in Supplementary materials and methods.

In brief, DNA was extracted from whole LN tissue sections of FL or microdissected BCL2‐positive follicles from LNs involved by ISFN. The rearranged IG genes and *BCL2–IGHJ* fusion were amplified from the FL samples using the respective BIOMED‐2 assays [1] and sequenced using the Illumina MiSeq method [10, 11].

In four cases, a clone‐specific PCR (CS‐PCR) was designed using a primer targeting the unique V(D)J sequence of the clonal *IGH/IGK* rearrangement, or the *BCL2–IGHJ* fusion. CS‐PCR was then used to screen the FL clonally related cells in ISFNs.

In addition, BaseScope *in situ* hybridisation (BS‐ISH) (Advanced Cell Diagnostics, Newark, CA, USA) using DNA probes specific to the unique VDJ or *BCL2–IGHJ* junctional sequence was performed to depict the localisation of FL clonally related cells.

Somatic mutations in FL were investigated by targeted sequencing of 70 genes (Agilent Technologies, Santa Clara, CA, USA) [12, 13]. These mutations were then investigated in ISFN samples using Fluidigm PCR (Fluidigm Access Array System, South San Francisco, CA, USA) and Illumina sequencing (Illumina, San Diego, CA, USA) [11, 12].

## Results

### Widespread ISFNs in LNs removed from solid neoplasia surgery

**Figure 1:**
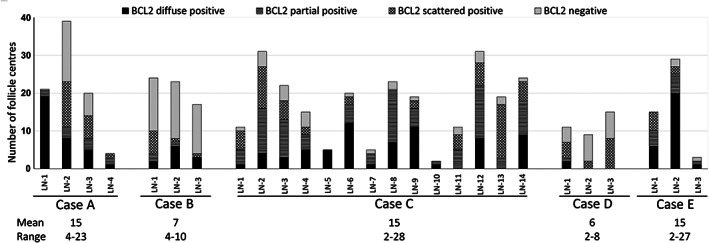
The extent of ISFN involvement in lymph nodes (LNs) removed from solid neoplasia surgery. Variations of BCL2 staining in various LNs that show prominent ISFN involvement as defined above (see Figure 3 for examples). The mean and range number of follicles involved by ISFN in each case are indicated. Case F has been excluded from the analysis as the slides available for research were not evaluable. Data includes only the LNs that show prominent ISFN involvement with at least one follicle displaying clustered or diffuse BCL2 positivity, and those with minimal involvement (few scattered strong BCL2‐positive cells in a germinal centre) are not included.

Several LNs cleared from the solid neoplasia surgery in cases A–E showed clear evidence of ISFN involvement by BCL2 immunohistochemistry, with one or more follicles displaying clustered or diffuse BCL2 positivity (Table 1 and Figure 1). Most of the remaining LNs in each of these cases also displayed some scattered strong BCL2‐positive cells in follicle centres, with staining intensity much higher than that of germinal centre T‐cells, suggesting potential involvement by ISFN cells (data not included in Figure 1 and Table 1).

### Clonal relationship between ISFNs and overt‐FL by CS‐PCR

Since the DNA quality from ISFN samples was poor (only amenable for optimal amplification of up to 200 bp genomic fragments), inadequate for conventional PCR of the rearranged IG genes and *BCL2–IGHJ* fusion, we first investigated the overt‐FL by PCR and sequencing of the clonally rearranged IG genes and *BCL2–IGHJ* fusion. We then designed CS‐PCR using a primer targeting the unique V(D)J (cases A and B: *IGH* and *IGK*, respectively) or *BCL2–IGHJ* (cases C and D) junction sequence. CS‐PCR sensitivity and specificity were attested using serial dilutions of the corresponding overt‐FL DNA and a range of unrelated lymphoid specimens (supplementary material, Figures S1 and S3).

**Figure 2:**
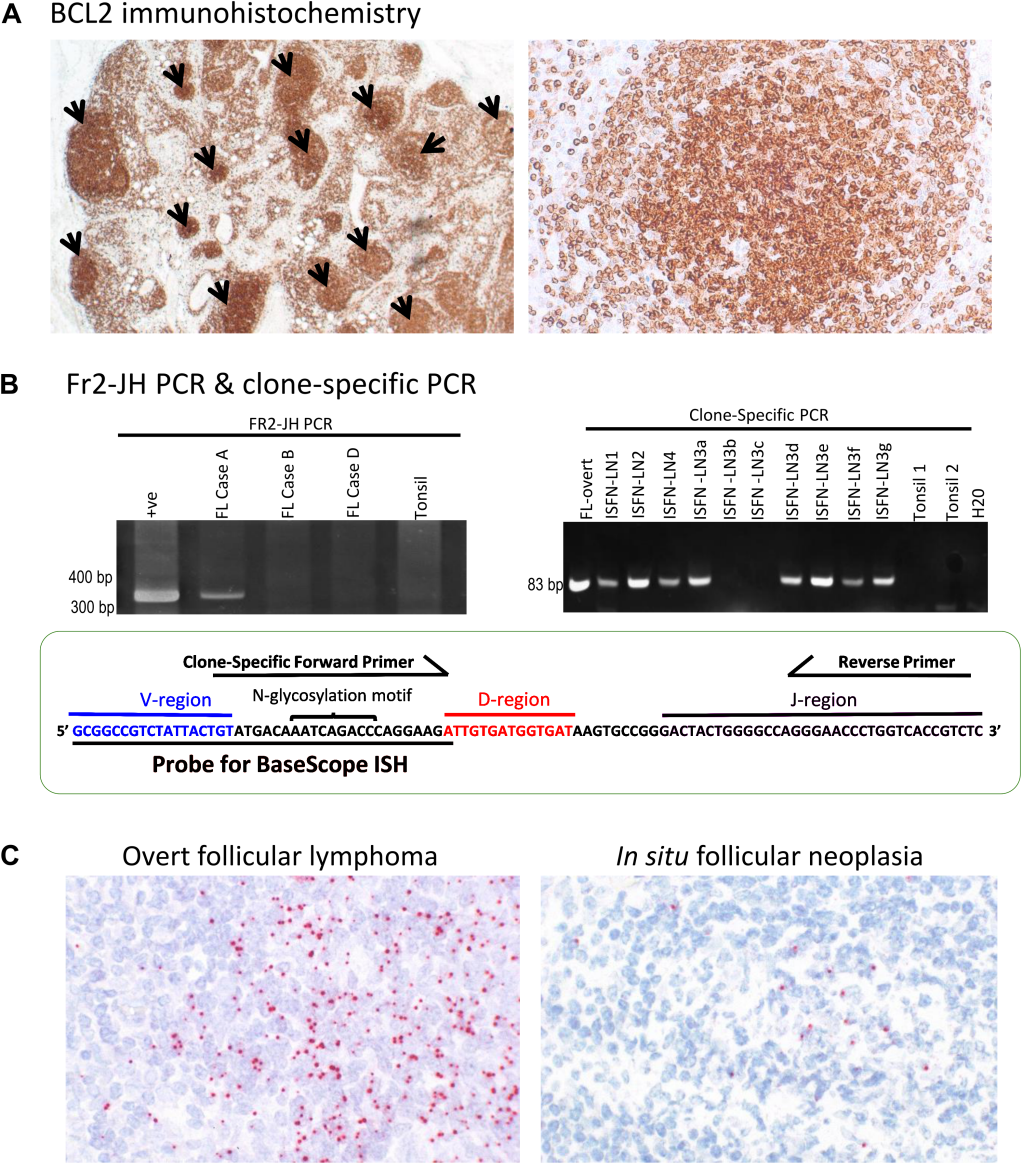
Confirmation of clonal identity between the early *in situ* follicular neoplasias (ISFNs) and late overt follicular lymphoma (FL) in case A. (A) BCL2 immunohistochemistry shows multiple ISFN lesions (indicated by arrows) in one of the involved lymph nodes (left panel) and strong BCL2 staining in the neoplastic cells of ISFN (right panel). (B) IG gene rearrangement analysis by BIOMED‐2 FR2‐JH PCR demonstrates a clonal *IGH* rearrangement (left panel). The CDR3 sequence is shown in the bottom panel, with the sequences used for designing the clone‐specific primer and BaseScope probe indicated. The sequence encoding an *N*‐glycosylation motif (N‐X‐S/T) is indicated [AAT: asparagine (N); CAG: glutamine (Q); and ACC: threonine (T)]. Examples of clone‐specific PCR are illustrated in the right panel. Multiple areas containing different follicles involved by ISFN in lymph node 3 (LN3) were microdissected and analysed (ISFN‐LN3a–g). (C) BaseScope *in situ* hybridisation shows diffuse hybridisation signals in a malignant follicle centre of overt‐FL (left panel) and scattered positivity in the follicle centre of an ISFN (right panel). Probe binding detected as red signal. Probe targets the end of the V‐region sequence, all of the V‐D junctional sequence, and the start of the D‐region sequence: 5'‐GCGGCCGTCTATTACTGTATGACAAATCAGACCCAGGAAGA‐3'. Any adjustments to the original image contrast to improve visualisation have been applied equally across the image.

**Figure 3:**
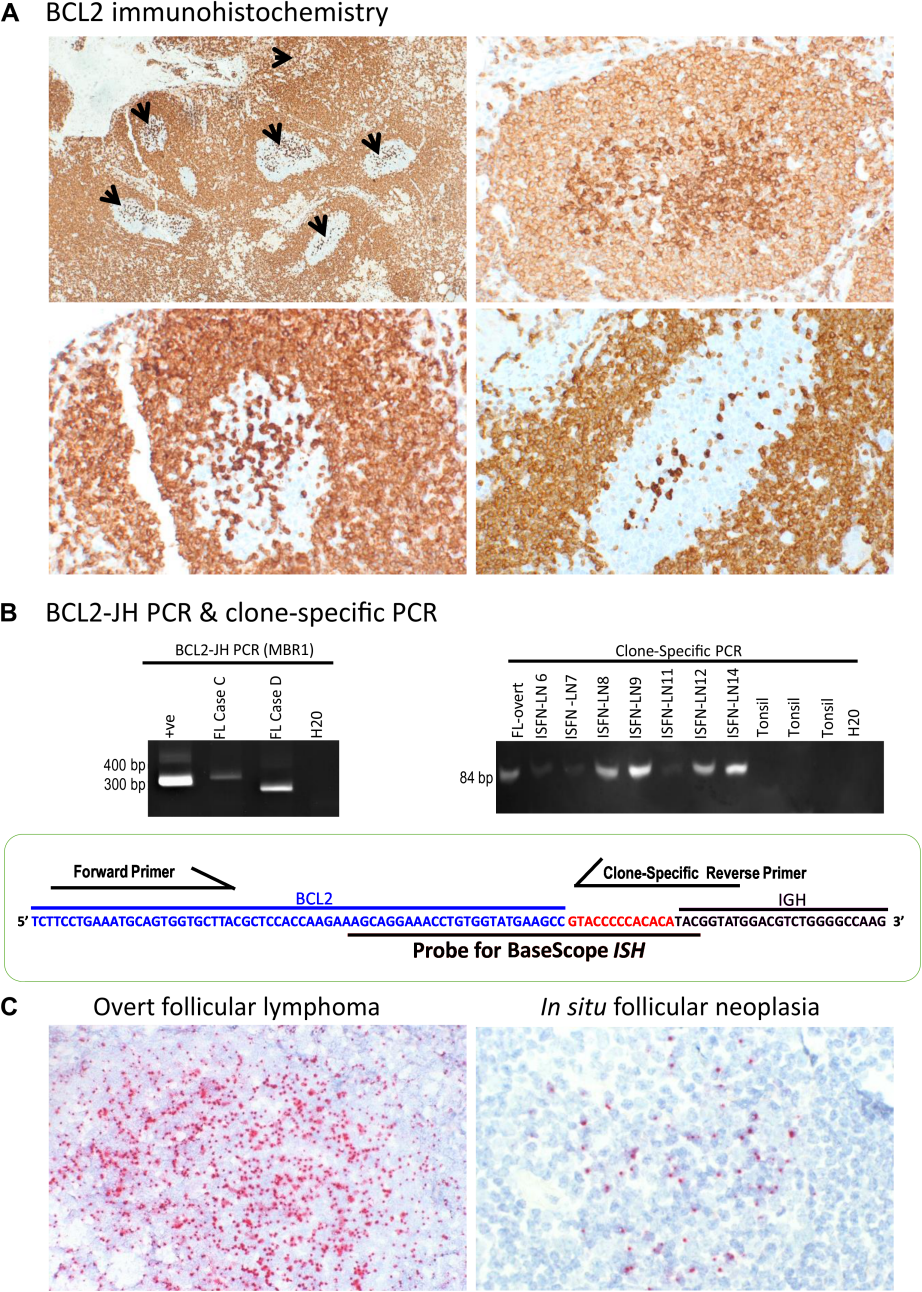
Confirmation of clonal identity between the early *in situ* follicular neoplasias (ISFNs) and late overt follicular lymphoma (FL) in case C. (A) BCL2 immunohistochemistry shows multiple ISFN lesions with strong BCL2 staining in the neoplastic ISFN cells (indicated by arrows) in one of the involved lymph nodes (top left panel), and diffuse (right panel), partial (bottom left) and scattered positivity (bottom right). (B) *BCL2*‐JH PCR by BIOMED‐2 assays (MBR1) shows a positive product (left panel). The *BCL2*‐*JH* fusion sequence is shown in the bottom panel, with the sequence used for designing the clone‐specific primer and BaseScope probe indicated. Examples of clone‐specific PCR are illustrated in the right panel. (C) BaseScope *in situ* hybridisation shows diffuse hybridisation signals in a malignant follicle centre of overt‐FL (left panel) and scattered positivity in the follicle centre of an ISFN (right panel). Probe binding detected as red signal. Probe targets the end of the *BCL2* gene sequence, all of the *BCL2–JH* fusion sequence, and the start of the *JH* sequence: 5'‐AAGCAGGAAACCTGTGGTATGAAGCCGTACCCCCACACATAC‐3'. Any adjustments to the original image contrast to improve visualisation have been applied equally across the image.

Next, we used CS‐PCR to investigate whether the FL clone was present in the ISFN LNs removed from the solid neoplasia surgery (Figures 2 and 3 and supplementary material, Figures S1–S3 and Table S1). We demonstrated the presence of the FL clone in almost all the LNs involved by ISFN in cases A (4/4), C (13/14), and D (4/4). In case B, we showed the presence of the FL clone in one involved LN but not in the two remaining nodes where ISFN was inconspicuous in the remaining tissue available for research.

The CDR3 sequencing analysis of case A also revealed an *N*‐glycosylation site.

### Clonal relationship between ISFN and overt‐FL by BS‐ISH

To prove and depict the clonal relationship among ISFNs and FL, we performed BS‐ISH in three cases. The probes were designed to bind the unique V‐D (case A) or *BCL2–IGH* (cases C and D) junction sequence. In each case, the specificity of BS‐ISH was ascertained by the expected hybridisation signals in the corresponding overt‐FL but not in unrelated lymphoid tissues. BS‐ISH identified the clonally related cells essentially in the follicle centre involved by ISFN in all LNs examined in each case (4/4 in case A, 6/6 in case C, and 3/3 in case D) (Figures 2 and 3 and supplementary material, Figure S2 and Table S1).

### Comparison of mutations between paired overt‐FL and ISFNs

**Figure 4:**
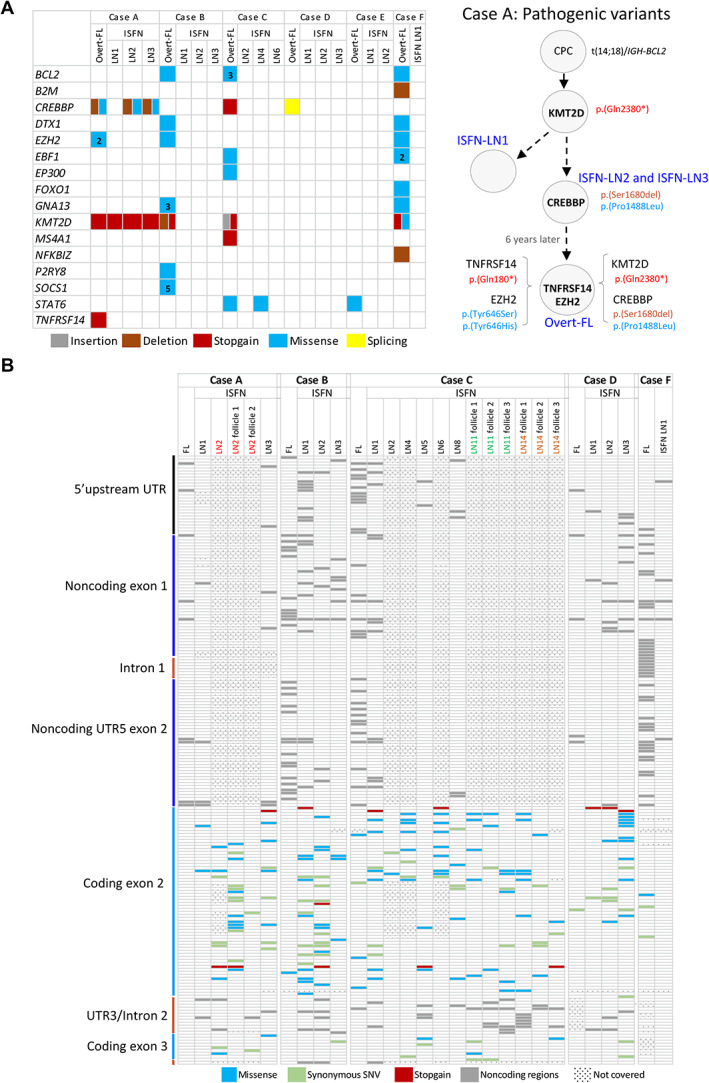
Comparison between pathogenic and *BCL2* variants identified in late overt follicular lymphoma (FL) and the matched early *in situ* follicular neoplasias (ISFNs). (A) Heatmap illustration of pathogenic variants identified in FL and ISFN. Left: summary of the predicted pathogenic variants identified in overt‐FL using a previously described target sequencing 70‐gene panel [12], and their detection in the ISFN‐involved LNs from the solid neoplasia surgery by Fluidigm PCR and Illumina sequencing. Grey: insertion; brown: deletion; red: stopgain; blue: missense; yellow: splicing; white: no variant detected. Right: predicted stepwise accumulation of pathogenic mutations for case A from common progenitor (CPC) to ISFN and overt‐FL. (B) Heatmap illustration of *BCL2* variants identified in FL and ISFN by Fluidigm PCR and Illumina sequencing. Analyses of lymph node (LN) involved by ISFN are based on pooled microdissected ISFN lesions, and also individual follicles involved by ISFN. The variants include all sequence changes in the region of *BCL2* gene sequenced from upstream of the 5'UTR to the end of the coding sequence of exon 3 (supplementary material, Table S4) and show remarkable divergence among ISFN LNs and overt‐FL. Variants were included based on cut‐off > 1% variant allele frequency and present in >5 reads in each direction for coding regions, while present in >10 reads in each direction for non‐coding regions. Blue: missense; green: synonymous SNV; red: stopgain; grey: noncoding region. Dots indicate data not available due to poor coverage.

We first investigated overt‐FL by targeted sequencing of 70 genes [12, 13]. The mutations identified were then screened in the ISFN LNs using PCR and Illumina sequencing. We identified potential pathogenic mutations in *CREBBP*, *EZH2*, *KMT2D*, *TNFRS14*, and *STAT6* in overt‐FL, and demonstrated their variable presence in the corresponding ISFNs (Figure 4A and supplementary material, Figure S4 and Table S2). In case A, there is evidence of stepwise accumulation of the observed mutations in the ISFN–overt‐FL sequence (Figure 4A).

As *BCL2* translocation causes its high transcriptional activities, hence predisposition to mutation by the somatic hypermutation machinery [14, 15], we examined *BCL2* sequence changes regardless of their impact on protein coding (Figure 4B). Remarkably, there were considerable divergences in *BCL2* variants among different ISFN‐involved LNs in the four cases successfully investigated and among different ISFN lesions within the same LN, indicating ongoing intraclonal diversifications.

## Discussion

By comprehensive investigation of the LNs cleared from the solid neoplasia surgery in patients who subsequently developed an overt‐FL, our present study uncovers the widespread distribution of ISFN lesions, involving many of the cleared LNs. Despite such extensive multifocal ISFN lesions in the cleared LNs, these ISFNs in each case are clonally related and linked to the subsequent overt‐FL. Remarkably, such widespread ISFN lesions occurred in middle‐aged individuals (45–57 years), and they remain dormant for a considerable period (4–16 years) before overt‐FL diagnosis.

Although the overall risk of t(14:18)/*IGH*::*BCL2* cells undergoing malignant transformation is low, a high level of their presence in the peripheral blood of healthy individuals is a predictive marker for FL development [16]. The widespread ISFNs in the cases investigated in the present study may also represent a relatively high clonal burden of t(14:18)/*IGH*::*BCL2*‐positive cells. However, the retrospective nature of the present study does not allow direct comparison with previous observations based on peripheral blood samples. Assessing the risk of FL development based on the extent of ISFN involvement would be a formidable challenge, given the large number of cases required.

Our findings indicate that the neoplastic cells of ISFN are actively trafficking, transiting from one B‐cell follicle to another and spreading widely among LNs. Like germinal centre B‐cells, the ISFN cells show a high level of intraclonal variations in their rearranged IG genes, particularly among different LNs, as shown by previous case studies [17, 18]. In line with this, our present study further demonstrates considerable sequence variations in the *BCL2* gene among different ISFN lesions including those from the same LN, most likely caused by the somatic hypermutation machinery [14, 15]. In keeping with the above observations, dynamic trafficking of BCL2‐overexpressing B‐cells and their multiple germinal centre transits have been elegantly documented in a mouse model study [18].

Comparison of the observed pathogenic mutations and *BCL2* variants reveals evidence of the co‐existence of different subclones in the same LN involved by ISFN (Figure 4). For example, the majority of the *BCL2* variants detected in the ISFN‐involved LNs for cases A and C were not found in overt‐FL, despite sharing *CREBBP/KMT2D* or *STAT6* mutations. There are three possible explanations: (1) these *BCL2* variants are artefacts from the experimental system. However, this is unlikely as they are reproducible in two experimental replicates, with some being common to different ISFN lesions; (2) these variants are from reactive B‐cells. Again, this is unlikely as BCL2 is not expressed in germinal centre B‐cells, thus not targeted by the hypermutation machinery [14, 19, 20]; (3) these variants are present in a subclone(s) of ISFN but not in those that eventually progressed into FL. The third possibility is the most pertinent, and this further underscores the dynamic nature of ISFN.

The protracted clonal expansion of ISFN cells in a germinal centre microenvironment may predispose them to a high risk of acquiring genetic changes that confer oncogenic potential, which are not efficiently repaired or eliminated due to apoptosis evasion by BCL2 overexpression. As expected, we confirm the variable presence of the lymphoma‐associated mutations in the ISFN lesions, although not to the extent reported previously [8, 9, 21]. Interestingly, the *CREBBP* mutation (p.S1680del) identified in case A, previously reported in a case with synchronous ISFN/DLBCL [9], and the *STAT6* mutation (p.D419) identified in cases C and E are the known hotspot changes in FL [22]. Nonetheless, ISFN lesions generally show no or few concurrent secondary mutations, in keeping with their insidious clinical and histological presentations.

Although broadly similar to the observations of previous studies [8, 9, 21], the present study showed a lesser extent of mutations in ISFN, notably by lack of the mutations associated with paired FL in four cases. Such variations are perhaps expected, due to the small number of cases investigated in each of these studies, and absence of detectable pathogenic mutations in ISFN was also seen in previous studies [8, 9, 21, 23], particularly in cases where paired FL/high‐grade B‐cell lymphoma lacked the mutations seen in ISFNs [8, 9]. Of note, the FL of cases D and E in the present study showed a paucity of typical FL mutations. In addition, a high proportion of ISFNs investigated in previous studies were identified synchronously with FL or high‐grade B‐cell lymphoma [7, 8, 9, 19], and these lesions are at a relatively ‘late phase’ of ISFN evolution. As t(14;18)/*IGH::BCL2*‐positive cells are actively trafficking, transiting from one follicle to another and in the meanwhile accumulating genetic changes, as evidenced by *BCL2* variants, the extent of genetic changes in ISFN may depend on the number of follicles that the t(14;18)/*IGH::BCL2* neoplastic cells have gone through, hence their phase during ISFN evolution. In line with this, Mamessier *et al* have shown progressive acquisition and accumulation of genetic changes from ISFNs to partial involvement by FL and overt‐FL, although based on non‐paired cases [21].

The quality of DNA samples from ISFNs in the present study is suboptimal, not permitting a large scale of mutation screening. Consequently, we only focused on analysis of the mutations identified in their paired FL. This would underestimate any potential mutations exclusively associated with ISFNs but not their paired FL. Nonetheless, the sequence regions containing mutations found in paired FL were adequately covered in ISFNs by PCR and next‐generation sequencing (NGS). The detection of FL‐associated mutations in paired ISFNs in cases A and C, and *BCL2* variants in ISFNs of all the cases ascertain the adequacy of the methodology used in the study.

In summary, our findings demonstrate the widespread distribution of ISFN lesions, further implicating their dynamic and fluidic nature with the neoplastic cells undergoing active trafficking and clonal evolution.

## Author contributions statement

RD, SL, FC, ZC and YT designed the experiments, collected data, and carried out the analysis. AW was responsible for case contribution and pathology. MQD and RD wrote and prepared the manuscript. MQD and AW designed and coordinated the study. All the authors commented on the manuscript and approved its submission for publication.

## Supporting information


Supplementary materials and methods

**Figure S1.** Confirmation of clonal identity between the early *in situ* follicular neoplasias (ISFNs) and late overt follicular lymphoma (FL) in case B
**Figure S2.** Confirmation of clonal identity between the early *in situ* follicular neoplasias (ISFNs) and late overt follicular lymphoma (FL) in case D
**Figure S3.** Analysis of the sensitivity and specificity of clone‐specific (CS) PCR
**Figure S4** Examples of somatic mutations identified by PCR and Illumina MiSeq sequencing in overt‐FL and matched ISFN lesion in case A (panels A–C) and case C (panel D)

**Table S1.** Summary of clonal identity results between lymph nodes from multiple ISFNs and subsequent matched overt‐FL
**Table S2.** Summary of the pathogenic variants identified in overt‐FL samples and matched ISFN samples
**Table S3.** Summary of unique clone‐specific (CS) PCR primers and conditions for cases A–D (referred to in Supplementary materials and methods)
**Table S4.** Summary of common sequence tagged primers used for targeted PCR sequencing in overt‐FL and matched ISFN for Illumina MiSeq
