# Supplementary material for: Widespread in situ follicular neoplasia in patients who subsequently developed follicular lymphoma
Source: J Pathol. 2022 Mar 3;256(4):369–77. doi: 10.1002/path.5861 (PMC9310836; doi:10.1002/path.5861)
Supplement: Supplementary file 1 — Supplementary materials and methods [file PATH-256-369-s003.docx]

**Widespread *in situ* follicular neoplasia in patients who subsequently developed follicular lymphoma**

R Dobson *et al. J Pathol* DOI: 10.1002/path.5861

**Supplementary materials and methods**

Reference numbers refer to the main text list

*Assessment of the extent of* in situ *follicular lymphoma* (*ISFN*) *involvement*

The extent of ISFN involvement in each lymph node was assessed by quantifying B-cell follicles according to BCL2 immunohistochemistry staining (diffuse, partial, scattered positive or absence of staining in germinal centre B-cells) (Figure 1B). BCL2 immunohistochemistry was carried out using H2(20) pre-treatment and a pre-diluted ready to use BCL2 antibody [clone: bcl-2/100/D5, Leica (Wetzlar, Germany)] on a Bond-III automated staining system.

*Tissue microdissection and DNA extraction*

The formalin‐fixed, paraffin‐embedded (FFPE) lymph node was available in each case. FFPE slides were reviewed by pathologists. Most of the overt-FL tissue did not require microdissection, due to a high number of tumour cells. Crude microdissection was performed on ISFN samples to enrich for BCL2-positive cells. Purified DNA was extracted using a QIAamp DNA Micro-Kit (Qiagen, Crawley, UK) from whole tissue sections of FL or BCL2-positive follicles from lymph nodes with ISFN. Purified DNA quality was assessed by PCR of variably sized genomic fragments (200, 300, 400, 600 bp) using a standardised protocol [11,12]. The DNA quality of the samples is summarised in supplementary material, Table S1. All FL samples, except case E, could amplify products of 300–400 bp. Case E overt-FL could amplify only 200 bp amplicons. DNA quality was tested on the microdissected tissue for ISFN samples, mostly amplifying fragments of up to 200–300 bp.

DNA was extracted from single follicles using a crude DNA extraction method, used due to small tissue quantities. Crude DNA extraction was carried out using overnight digestion of microdissected tissue with Proteinase K and NP-40 at 56 °C. Therefore, DNA quality was not analysed for DNA extracted from single follicle areas.

*PCR and sequencing of the rearranged* IG *genes and* BCL2–IGH *fusion*

The rearranged *IGH*, *IGK*, and *IGL* genes, and *BCL2–IGH* genomic fusion were amplified using the respective BIOMED‐2 method [1], with the primers tagged with a common sequence (CS1: 5'‐ACACTGACGACATGGTTCTACA‐3′ or CS2: 5'‐TACGGTAGCAGAGACTTGGTCT‐3′) to enable Illumina sequencing. All PCRs were performed in duplicate. PCRs were carried out using a FastStart High Fidelity PCR system (Roche, Basel, Switzerland). The amplified products were routinely analysed by electrophoresis on 6–8% polyacrylamide gels. Positive PCR products were purified using AMPure XP beads (Beckman Coulter, Pasadena, CA, USA), barcoded, and pooled for Illumina MiSeq V3 sequencing as previously described [10,11]. In addition, the *BCL2–IGH* fusion PCR products were sequenced by Illumina MiSeq V3 sequencing and the Sanger sequencing method.

The IG sequence data were first analysed using the Vidjil software (VidjilNet Consortium, Lille, France) (http://[www.vidjil.org](http://www.vidjil.org)) [24] to identify the clonal IG gene rearrangement. The V(D)J joining sequence of the clonal IG gene rearrangement was then delineated using IMGT/V-Quest (http://imgt.org) (ImMunoGeneTics information system, Montpellier, France) and IgBlast (<https://www.ncbi.nlm.nih.gov/igblast/>) (NCBI, Bethesda, MD, USA).

*BCL2–IGH* fusion sequences were analysed using the IGV software ([www.broadinstitute.org/software/igv/](http://www.broadinstitute.org/software/igv/)) (Broad Institute, Cambridge, MA, USA), and the unique junctional sequence between the *BCL2* and *IGH* genes was identified using BLAT/BLAST analysis (http://[www.ensembl.org](http://www.ensembl.org)).

*Clone-specific PCR* (*CS-PCR*)

In each case, a clone-specific primer was designed according to the unique V(D)J sequence of clonal IGH (case A)/IGK (case B) rearrangement or the *BCL2–IGH* fusion (cases C and D) identified from FL. This, together with a primer targeting either upper or downstream sequence, formed a strategy for CS-PCR (CS-PCR primers are summarised in supplementary material, Table S3). CS-PCRs were carried out using the FastStart High Fidelity PCR system (Roche).

The conditions for CS-PCR were optimised in each case using the corresponding FL as positive and reactive tonsils as negative control. The optimised cycling conditions are also summarised in supplementary material, Table S3. The sensitivity of each CS-PCR was determined by using serial dilutions of the respective overt-FL samples. Once conditions were optimised, CS-PCR was carried out using DNA extracted from ISFN. PCR products were routinely analysed by electrophoresis on 10–12% polyacrylamide gels and representative amplicons were confirmed by Sanger sequencing.

*BaseScope* in situ *hybridisation* (*ISH*)

Specific DNA probes were designed to target the unique V-D (case A) or *BCL2–IGH* (cases C and D) junctional sequence identified from FL and then used to identify the related clonal B-cells by BaseScope *in situ* hybridisation. This was carried out according to the manufacturer’s instructions (Advanced Cell Diagnostics, Newark, CA, USA) and experimental protocols were optimised by systematically testing protease K digestion, target retrieval, and hybridisation conditions as described previously [10].

*Mutation analysis of FL by targeted sequencing*

DNA samples from FL were subjected to targeted sequencing of 70 genes (previously described by Cucco *et al* [12,13]), which were frequently mutated in FL and diffuse large B-cell lymphoma. This was carried out using HaloPlex HS target enrichment (Agilent Technologies, Santa Clara, CA, USA) and Illumina Hiseq sequencing as previously described [12,13], with FL samples for cases A and C investigated in duplicate due to suboptimal DNA quality. The FL sample for case E was not included for HaloPlex target enrichment due to its inadequate DNA quality (200 bp). The sequence data analysis, variant calling, and filtering were performed as described in our previous studies [12,13].

*Mutation analysis of ISFN lesions by targeted sequencing*

The DNA quality from most lymph nodes containing ISFN was inadequate for the above panel sequencing using the HaloPlex HS target enrichment. To investigate whether the mutations identified in FL were present in the paired ISFN lesions, we adopted the Fluidigm PCR-based target enrichment (Fluidigm Access Array System, South San Francisco, CA, USA) using cycling conditions previously described [11]. To enable PCR of suboptimal DNA samples from the ISFN lesions, the primers were designed to span a short stretch (105–215 bp) of DNA sequences. Each DNA sample was investigated at least in duplicate. The experiment, variant calling, and filtering were as previously described [11,12]. Since the load of clonal B-cell population in ISFN-involved lymph nodes was relatively low, variants with a variant allele frequency (VAF) ≥ 1% and minimal alternative allele reads ≥ 5 from each sequencing direction in both replicates were considered as true change. In addition, the Fluidigm PCR-based method was used to amplify and sequence the *BCL2* gene, covering from upstream of the 5'UTR along to the end of the coding sequence of exon 3. The primers used are summarised in supplementary material, Table S4.
