# Supplementary material for: Widespread in situ follicular neoplasia in patients who subsequently developed follicular lymphoma
Source: J Pathol. 2022 Mar 3;256(4):369–77. doi: 10.1002/path.5861 (PMC9310836; doi:10.1002/path.5861)
Supplement: Supplementary file 2 — Figure S1. Confirmation of clonal identity between the early in situ follicular neoplasias (ISFNs) and late overt follicular lymphoma (FL) in case B Figure S2. Confirmation of clonal identity between the early in situ follicular neoplasias (ISFNs) and late overt follicular lymphoma (FL) in case D Figure S3. Analysis of the sensitivity and specificity of clone‐specific (CS) PCR Figure S4 Examples of somatic mutations identified by PCR and Illumina MiSeq sequencing in overt‐FL and matched ISFN lesion in case A (panels A–C) and case C (panel D) [file PATH-256-369-s002.docx]

**Widespread *in situ* follicular neoplasia in patients who subsequently developed follicular lymphoma**

R Dobson *et al. J Pathol* DOI: 10.1002/path.5861

**Supplementary Figures S1–S4**


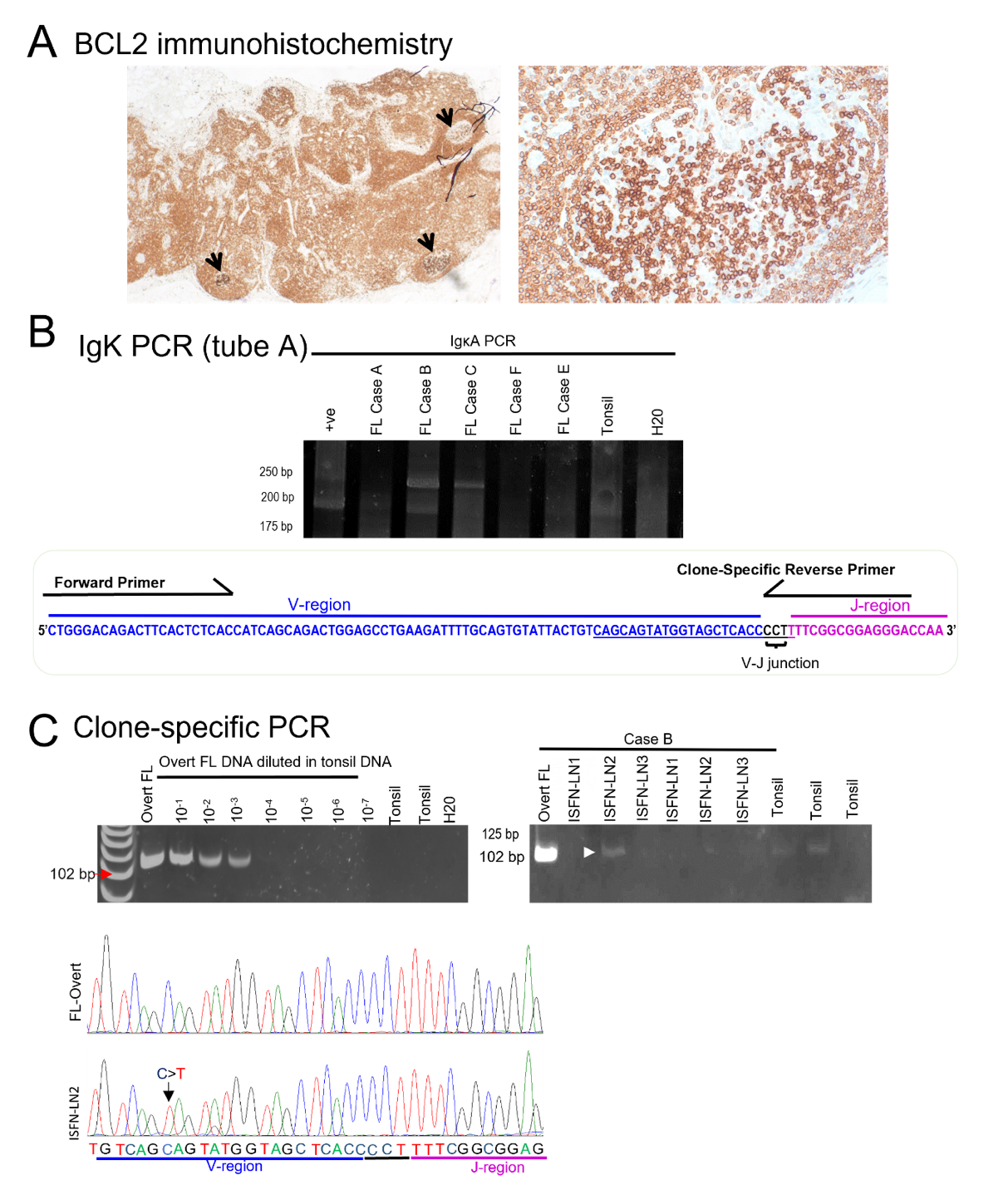


**Figure S1.** Confirmation of clonal identity between the early *in situ* follicular neoplasias (ISFNs) and late overt follicular lymphoma (FL) in case B. (A) BCL2 immunohistochemistry shows multiple ISFN lesions (indicated by arrows) in one of the involved lymph nodes (left panel) and strong BCL2 staining in the neoplastic cells of ISFN (right panel). (B) IG$\kappa$ gene rearrangement analysis by BIOMED-2 PCR demonstrates a clonal IG$\kappa$rearrangement (upper panel). The CDR3 sequence was used for designing the clone-specific primer indicated (bottom panel). (C) Clone-specific (CS) PCR: sensitivity testing using serial dilution of overt-FL DNA sample (left panel) and examples of CS-PCR (right panel, with positive indicated by arrowhead). Bottom panel: examples of Sanger sequencing to confirm CS-PCR products. Any adjustments to the original image contrast to improve visualisation have been applied equally across the image.

**Figure S2.** Confirmation of clonal identity between the early *in situ* follicular neoplasias (ISFNs) and late overt follicular lymphoma (FL) in case D. (A) BCL2 immunohistochemistry shows multiple ISFNs (indicated by arrows) in one of the involved lymph nodes (left panel) and strong BCL2 staining in the neoplastic cells of a follicle involved by ISFN (right panel). (B) *BCL2-JH* PCR by BIOMED-2 assays (MBR1) shows a positive product (please refer to Figure 3B) and Sanger sequencing identifies the unique junction sequence, with the region used for designing the clone-specific (CS) primer and BaseScope probe indicated (bottom panel). Examples of CS-PCR and Sanger sequencing to confirm CS-PCR products are illustrated (top panel). (C) BaseScope *in situ* hybridisation shows diffuse hybridisation signals in a malignant follicle centre of overt FL (left panel) and scattered positivity in the follicle centre of an ISFN (right panel). Probe detected as red signal. Any adjustments to the original image contrast to improve visualisation have been applied equally across the image.

**Figure S3.** Analysis of the sensitivity and specificity of clone-specific (CS) PCR. Example of CS-PCR assay sensitivity determined by PCR using serial dilutions of overt-FL into tonsil DNA (left panels) in cases A (panel A), C (panel B), and D (panel C), and representative Sanger sequencing confirmation of CS-PCR products (right panels). Any adjustments to the original gel image contrast to improve visualisation have been applied equally across the image.

 **Figure S4.** Examples of somatic mutations identified by PCR and Illumina MiSeq sequencing in overt-FL and matched ISFN lesion in case A (panels A–C) and case C (panel D). Aligned reads were transformed to a bam file and visualised using IGV software.
