# Supplementary material for: Widespread in situ follicular neoplasia in patients who subsequently developed follicular lymphoma
Source: J Pathol. 2022 Mar 3;256(4):369–77. doi: 10.1002/path.5861 (PMC9310836; doi:10.1002/path.5861)
Supplement: Supplementary file 3 — Table S1. Summary of clonal identity results between lymph nodes from multiple ISFNs and subsequent matched overt‐FL Table S2. Summary of the pathogenic variants identified in overt‐FL samples and matched ISFN samples Table S3. Summary of unique clone‐specific (CS) PCR primers and conditions for cases A–D (referred to in Supplementary materials and methods) Table S4. Summary of common sequence tagged primers used for targeted PCR sequencing in overt‐FL and matched ISFN for Illumina MiSeq [file PATH-256-369-s001.docx]

**Widespread *in situ* follicular neoplasia in patients who subsequently developed follicular lymphoma**

R Dobson *et al. J Pathol* DOI: 10.1002/path.5861

**Supplementary Tables S1–S4**

**Table S1.** Summary of clonal identity results between lymph nodes from multiple ISFNs and subsequent matched overt-FL

| **Case** | **Sample name** | **DNA quality (amplifiable fragment size in bp)** | **Clone-specific**  **PCR result*** | **Confirmation by Sanger sequencing** | **Confirmation by BaseScope ISH** |
| --- | --- | --- | --- | --- | --- |
| A | Overt-FL | 400 (faint) | Positive | Yes | Yes |
|  | ISFN-LN1 | 200 | Positive | Yes | Yes |
|  | ISFN-LN2 | 200 | Positive | Yes | Yes |
|  | ISFN-LN3 | 200 (combined and micro-dissected regions A, D, E, F, G), while micro-dissected regions 3B and 3C <200 bp | Positive | Yes | Yes |
|  | ISFN-LN4 | 200 | Positive | Yes | Yes |
| B | Overt-FL | 400 | Positive | Yes | – |
|  | ISFN-LN1 | 200 | Failed | – | – |
|  | ISFN-LN2 | 200 | Positive | Yes | – |
|  | ISFN-LN3 | 200 | Failed | – | – |
| C | Overt-FL-LN1 | 300 | Positive | Yes | – |
|  | Overt-FL-LN2 | 300 | Positive | Yes | Yes |
|  | Overt-FL-LN3 | 200 | Positive | – | – |
|  | ISFN-LN1 | 300 faint | Positive | – | Yes |
|  | ISFN-LN2 | 300 faint | Positive | – | – |
|  | ISFN-LN3 | 300 faint | Positive | – | – |
|  | ISFN-LN4 | 300 | Positive | – | – |
|  | ISFN-LN5 | 200 | Positive | – | Yes |
|  | ISFN-LN6 | 300 faint | Positive | Yes | – |
|  | ISFN-LN7 | 300 faint | Positive | Yes | – |
|  | ISFN-LN8 | 300 faint | Positive | – | Yes |
|  | ISFN-LN9 | 300 faint | Positive | – | Yes |
|  | ISFN-LN10 | 300 faint | Failed | – | – |
|  | ISFN-LN11 | 300 faint | Positive | – | – |
|  | ISFN-LN12 | 300 | Positive | Yes | Yes |
|  | ISFN-LN13 | 300 | Positive | – | – |
|  | ISFN-LN14 | 200 | Positive | – | Yes |
| D | Overt-FL | 400 | Positive | Yes | Yes |
|  | ISFN-LN1 | 200 | Positive | – | Yes |
|  | ISFN-LN2 | 200 | Positive | Yes | Yes |
|  | ISFN-LN3 | 200 | Positive | Yes | Yes |
|  | ISFN-LN4 | 200 | Positive | Yes | – |
| E | Overt-FL | <200 | – | – | – |
|  | ISFN-LN1 | 300 | – | – | – |
|  | ISFN-LN2 | 200 | – | – | – |
|  | ISFN-LN3 | 300 faint | – | – | – |
| F | Overt-FL | 400 faint | – | – | – |
|  | ISFN-LN1 | 400 faint | – | – | – |

FL: follicular lymphoma; ISFN: *in situ* follicular neoplasia; ‘–‘ denotes result not available

*Clone-specific PCR was performed using specific primer to the CDR3 region of the rearranged IG heavy chain (case A) and Igκ light chain genes (case B), respectively, or the BCL2–JH fusion junctional sequence (cases C and D).

DNA quality was determined using a quality control PCR method (previously described by Cucco *et al* [12]) to determine the maximum fragment sizes amplified.

**Table S2.** Summary of the pathogenic variants identified in overt-FL samples and matched ISFN samples.

The variants present in overt-FL samples were initially identified using 70-gene panel HaloPlex target sequencing. The presence of the identified pathogenic variants was then explored using PCR approach and Illumina MiSeq to confirm if the variants were present in the FL and matched ISFN. Samples with ≥1% variant allele frequency (VAF) and present in ≥5 reads in each direction in at least 2 duplicates were classed as ‘present’. ‘–‘indicates that the variant was not identified as present in that sample based on the cut-off criteria.

| **Case A** | | | | | | | |
| --- | --- | --- | --- | --- | --- | --- | --- |
| **Gene** | **Variant** | **cDNA change** | **Protein change** | **Overt-FL** | **ISFN-LN1** | **ISFN-LN2** | **ISFN-LN3** |
| *CREBBP* | chr16:3731323_AGG>- | c.5039_5041del | p.1680_1681del | Present (VAF: 36.8%) | ─ | Present (VAF: 7.75%) | Present (VAF: 2.95%) |
| *CREBBP* | chr16:3736747_G>A | c.C4463T | p.P1488L | Present (VAF: 38.1%) | – | Present (VAF: 31.48%) | Present (VAF: 12.4%) |
| *EZH2* | chr7:148811635_T>G | c.A1937C | p.Y646S | Present (VAF: 22.6%) | ─ | ─ | ─ |
| *EZH2* | chr7:148811636_A>G | c.T1936C | p.Y646H | Present (VAF: 9.75%) | ─ | ─ | ─ |
| *KMT2D* | chr12:49040632_G>A | c.C7138T | p.Q2380X | Present (VAF: 71.2%) | Present (VAF: 18.38%) | Present (VAF: 12.87%) | Present (VAF: 8.43%) |
| *TNFRSF14* | chr1:2560701_C>T | c.C538T | p.Q180X | Present (VAF: 71.1%) | ─ | ─ | ─ |
| **Case B** | | | | | | | |
| **Gene** | **Variant** | **cDNA change** | **Protein change** | **Overt-FL** | **ISFN-LN1** | **ISFN-LN3** | **ISFN-LN2** |
| *BCL2* | chr18:63318094_A>T | c.T573A | p.D191E | Present (VAF: 20%) | ─ | ─ | ─ |
| *DTX1* | chr12:113058384_G>C | c.G192C | p.Q64H | Present (VAF: 19%) | ─ | ─ | ─ |
| *EZH2* | chr7:148811635_T>G | c.A1937C | p.Y646S | Present (VAF: 26.5%) | ─ | ─ | ─ |
| *GNA13* | chr17:65056368_A>G | c.T226C | p.F76L | Present (VAF: 18.5%) | ─ | ─ | ─ |
| *GNA13* | chr17:65056380_G>C | c.C214G | p.H72D | Present (VAF: 18.5%) | ─ | ─ | ─ |
| *GNA13* | chr17:65056513_C>G | c.G81C | p.Q27H | Present (VAF: 15.5%) | ─ | ─ | ─ |
| *KMT2D* | chr12:49027005_C>T | c.G14961A | p.W4987X | Present (VAF: 16.5%) | ─ | ─ | ─ |
| *KMT2D* | chr12:49046352_GT>- | c.4490_4491del | p.H1497fs | Present (VAF: 15.5%) | ─ | ─ | ─ |
| *P2RY8* | chrX:1465922_A>T | c.T637A | p.Y213N | Present (VAF: 19.5%) | ─ | ─ | ─ |
| *SOCS1* | chr16:11255100_G>A | c.C379T | p.R127C | Present (VAF: 18%) | ─ | ─ | ─ |
| *SOCS1* | chr16:11255105_C>G | c.G374C | p.S125T | Present (VAF: 18.5%) | ─ | ─ | ─ |
| *SOCS1* | chr16:11255129_A>G | c.T350 | p.V117A | Present (VAF: 18%) | ─ | ─ | ─ |
| *SOCS1* | chr16:11255305_G>C | c.C174G | p.F58L | Present (VAF: 30.5%) | ─ | ─ | ─ |
| *SOCS1* | chr16:11255373_G>C | c.C106G | p.P36A | Present (VAF: 31.5%) | ─ | ─ | ─ |
| **Case C** | | | | | | | |
| **Gene** | **Variant** | **cDNA change** | **Protein change** | **Overt-FL** | **ISFN-LN2** | **ISFN-LN4** | **ISFN-LN6** |
| *BCL2* | chr18:63318151_G>T | c.C516A | p.N172K | Present (VAF: 40%) | ─ | ─ | ─ |
| *BCL2* | chr18:63318542_G>A | c.C125T | p.A42V | Present (VAF: 36%) | ─ | ─ | ─ |
| *BCL2* | chr18:63318063_C>G | c.G604C | p.V202L | Due to presence of artefact in some reads, the true VAF cannot be determined. However, it is clear from Bam files that this variant is present in overt-FL (as confirmed with HaloPlex sequencing analysis at 21% VAF) but not in any of the matched ISFN samples | | | |
| *CREBBP* | chr16:3773903_G>A | c.C2311T | p.Q771X | Present (VAF: 36%) | ─ | ─ | ─ |
| *EBF1* | chr5:159097078_G>C | c.C187G | p.R63G | Present (VAF: 34.5%) | ─ | ─ | ─ |
| *EP300* | chr22:41170455_T>G | c.T4336G | p.Y1446D | Present (VAF: 37.5%) | ─ | ─ | ─ |
| *KMT2D* | chr12:49029468_C>A | c.G14008T | p.E4670X | Present (VAF: 33.5%) | ─ | ─ | ─ |
| *KMT2D* | chr12:49040662_->A | c.7107dupT | p.R2370fs | Present (VAF: 23.5%) | ─ | ─ | ─ |
| *MS4A1* | chr11:60463100_C>A | c.C258A | p.Y86X | Present (VAF: 34.5%) | ─ | ─ | ─ |
| *STAT6* | chr12:57102879_C>T | c.G1255A | p.D419N | Present (VAF: 34%) | ─ | Present (VAF: 1.19%) | ─ |
| **Case D** | | | | | | | |
| **Gene** | **Variant** | **cDNA change** | **Protein change** | **Overt-FL** | **ISFN-LN1** | **ISFN-LN2** | **ISFN-LN3** |
| *CREBBP* | chr16:3757286_A>G | Splicing |  | Present (VAF: 11.5%) | ─ | ─ | ─ |
| **Case E** | | | | | | | |
| **Gene** | **Variant** | **cDNA change** | **Protein change** | **Overt-FL** | **ISFN-LN3** | **ISFN-LN2** | **N/A** |
| *STAT6* | chr12:57102878_T>G | c.A1256C | p.D419A | Present (VAF: 10.5%) | ─ | ─ | N/A |
| **Case F** | | | | | | | |
| **Gene** | **Variant** | **cDNA change** | **Protein change** | **Overt-FL** | **ISFN-LN1** | **N/A** | **N/A** |
| *B2M* | chr15:44711545_AGATGTCT>- | Whole gene |  | Present (VAF: 20%) | ─ | N/A | N/A |
| *BCL2* | chr18:63318329_G>C | c.C338G | p.A113G | Present (VAF: 18%) | ─ | N/A | N/A |
| *DTX1* | chr12:113095199_T>A | c.T1544A | p.I515N | Present (VAF: 20%) | ─ | N/A | N/A |
| *EBF1* | chr5:159073411_G>T | c.C539A | p.P180Q | Present (VAF: 22.5%) | ─ | N/A | N/A |
| *EBF1* | chr5:159073412_G>T | c.C538A | p.P180T | Present (VAF: 23%) | ─ | N/A | N/A |
| *EZH2* | chr7:148811635_T>G | c.A1937C | p.Y646S | Present (VAF: 77.5%) | ─ | N/A | N/A |
| *FOXO1* | chr13:40665764_C>T | c.G449A | p.R150H | Present (VAF: 16.5%) | ─ | N/A | N/A |
| *GNA13* | chr17:65014525_A>T | c.T866A | p.F289Y | Present (VAF: 28%) | ─ | N/A | N/A |
| *KMT2D* | chr12:49022146_A>T | c.T16418A | p.I5473N | Present (VAF: 17.5%) | ─ | N/A | N/A |
| *KMT2D* | chr12:49033328_G>A | c.C11377T | p.Q3793X | Present (VAF: 15.5%) | ─ | N/A | N/A |
| *NFKBIZ* | chr3:101849804_CGCCCGGCT>- | c.176_184del | p.59_62del | Present (VAF: 23%) | ─ | N/A | N/A |

**Table S3.** Summary of unique clone-specific (CS) PCR primers and conditions for cases A–D.

Top table: CS-PCR primers are based on overt-FL IGH/BCL2-IGH sequencing results. Predicted Tm determined using Primer-Blast default conditions. Bottom table: optimised PCR touchdown cycling conditions for each CS-PCR assay (red indicates variable factors between cases)

| **CS-PCR primers** | | | | | | |
| --- | --- | --- | --- | --- | --- | --- |
| **Case** | **Sequence CS-primer based on** | **Forward primer** | **Predicted Tm (forward primers)** | **Reverse primer** | **Amplicon size (bp)** | **Predicted Tm (reverse primers)** |
| A | V-D junctional sequence from *IGH*-FR2 | 5' TGTATGACAAATCAGACCCAGGAAG 3' | 60.57 | 5' GAGACGGTGACCAGGGTTCC 3' | 83 | 62.17 |
| B | V-J junctional sequence from IGK Tube A | 5' CTGGGACAGACTTCACTCTCAC 3' | 60.03 | 5' TCCCTCCGCCGAAAAGG 3' | 102 | 58.92 |
| C | N-fusion region between translocated *IGH* and *BCL2* (MBR1) | 5' TTCCTGAAATGCAGTGGTGCTTA 3' | 60.75 | 5' CATACCGTATGTGTGGGGGTAC 3' | 84 | 60.22 |
| D | N-fusion region between translocated *IGH* and *BCL2* (MBR1) | 5' AACACAGACCCACCCAGAG 3' | 58.85 | 5' AGTAGTAGCCTAACCGGAGA 3' | 100 | 56.59 |
| **% DMSO** | Case A: 7%; cases B, C, and D: 10% | | | | | |
| **Optimised CS-PCR cycle conditions** | | | | | | |
| Temp (°C) | | | Time | Cycles | Phase | |
| 95 | | | 10 min | 1 | 1 | |
| 95 | | | 30 s | 2 | 2 (cases C and D skip this phase) | |
| Cases A and B: 68°C | | | 30 s |  |  |  |
| 72 | | | 1 min |  |  |  |
| 95 | | | 30 s | Case A: 2; cases B and C: 5; case D: 7 | 3 | |
| Touchdown PCR (reducing 1°C each cycle): case A: 67–66°C; case B: 67–63°C; case C: 67–63°C; case D: 67–60°C | | | 30 s |  |  |  |
| 72 | | | 1 min |  |  |  |
| 95 | | | 30 s | 38 | 4 | |
| Case A: 66°C; case B: 63°C; case C: 63°C; case D: 60°C | | | 30 s |  |  |  |
| 72 | | | 1 min |  |  |  |
| 72 | | | 5 min | 1 | 5 | |

**Table S4.** Summary of common sequence tagged primers used for targeted PCR sequencing in overt-FL and matched ISFN for Illumina MiSeq.

CS1: 5'‐ACACTGACGACATGGTTCTACA‐3'

CS2: 5'‐TACGGTAGCAGAGACTTGGTCT‐3'

| **Primer name** | **Primer sequence (5'–3')** | **Amplicon length (bp)** |
| --- | --- | --- |
| **BCL2 primers** | | |
| 1 (5' Upstream) | CTCCGGGCCCTCCCT | 120 |
|  | TGCGCACCCTTTCTCCTC |  |
| 2 (5' Upstream) | GCCGTAGCCAGCGCC | 123 |
|  | TGACTGCTACGAAGTTCTCCC |  |
| 3 (5' Upstream) | GGGAGAACTTCGTAGCAGTCA | 122 |
|  | GCCGAGCGCTAGAAGCC |  |
| 4 (Noncoding exon 1) | CACAGCGCGGGCTTCT | 128 |
|  | GGGACGGAGGCAGGAAT |  |
| 5 (Noncoding exon 1) | AAGAGGATTCCTGCCTCCGT | 144 |
|  | CCCTTCTCGGCAATTTACACG |  |
| 6 (Noncoding exon 1) | CGGTCCCGTGGATAGAGATTC | 129 |
|  | GCGGCGGCAGATGAATTAC |  |
| 7 (Noncoding exon 1) | GACTTCTGCGAATACCGGACT | 133 |
|  | TTCCCAGACTTCTGCTTCACA |  |
| 8 (Noncoding exon 1) | AGATCTCCGGTTGGGATTCCT | 125 |
|  | TTGAAACTTCCCAATGAATCAGGAG |  |
| 9 (Noncoding exon 1) | GCGACTCCTGATTCATTGGG | 128 |
|  | CAGCATGATCCTCTGTCAAGTTTC |  |
| 10 (Noncoding exon 1) | ATGGGATCGTTGCCTTATGC | 198 |
|  | AAGAATGTATTAAGCTGCCTGGAA |  |
| 11 (Exon 2) | AACCTTTCAGCATCACAGAGGA | 122 |
|  | CCTTGGCATGAGATGCAGG |  |
| 12 (Exon 2) | ACGTGCCTCATGAAATAAAGATCC | 130 |
|  | GCTTTGCATTCTTGGACGAGG |  |
| 13 (Exon 2) | AGAATCAAGTGTTCCGCGTG | 139 |
|  | GCCAACGGCACCTCTCG |  |
| 14 (Exon 2) | CGAGAGGTGCCGTTGGC | 120 |
|  | CCTCTGCGACAGCTTATAATGG |  |
| 15 (Exon 2) | AAGTACATCCATTATAAGNTGTCGC | 140 |
|  | CGGGATGCGGCTGGAT |  |
| 16 (Exon2) | CATCTTCTCCTCCCAGCC | 210 |
|  | CTGGACATCTCGNCGAAGT |  |
| 17 (Exon 2) | GACTTCTCCCGCCGCTAC | 110 |
|  | CTGAAGAGCTCCTCCACCAC |  |
| 18 (Exon 2) | TGGAGGAGCTCTTCAGGGAC | 130 |
|  | ATCCACAGGGCGATNTTGTC |  |
| 19 (Exon 2) | GGTCATGTGTGTGGAGAG | 148 |
|  | CAGACTCANATCACCAAGT |  |
| 20 (Exon 2) | CTGAGTACCTGAACCGGCAC | 153 |
|  | CTCCACAGCCTCCCATTGC |  |
| 21 (Exon 3) | CAGGATGCCTCTTTCTCT | 167 |
|  | CTGAGCAGAGTCTTCAGAG |  |
| 22 (Exon 3) | GCCTCTGTTTGATTTCTC | 110 |
|  | CATGTTGACTTCACTTGTG |  |
| 23 (Exon 3) | TGCCTATCTGGGCCACAAGT | 124 |
|  | ACAGCCTGCAGCTTTGTTTC |  |
| **B2M primers** | | |
| 1 | CGGGCCTTGTCCTGATT | 208 |
|  | GCAGAGCGGGAGAGGAAG |  |
| **BTG primers** | | |
| 1 | CCATCTGTGTGCTGTATGAAG | 194 |
|  | CCATCATCATCAGATGATCCATC |  |
| **CREBBP primers** | | |
| 1 | GCTTCCGAACTACAGCTCTGG | 189 |
|  | CCATGCCCACACTCATCG |  |
| 2 | AGACTTTGTGCTGCTATGGGA | 124 |
|  | ATGAACGTGCCTTGCCCTAA |  |
| 3 | TGTGTGCGTGGGTCCTG | 182 |
|  | TAGTCATGGATGATCCGCTCT |  |
| 4 | TGCTCAGCTGTGACCTCATG | 159 |
|  | TGCAGGTGTAGACAAAGCGG |  |
| **DTX1 primers** | | |
| 1a | GGAGTGGCTGAATGAGCAC | 196 |
|  | TGGGGTGGGTGTCTGC |  |
| 1b | GGAGTGGCTGAATGAGCAC | 143 |
|  | GGTCGATGATGTAGGGCACA |  |
| 2 | CGCCTGGGAAGATGGAGTTC | 163 |
|  | GCATCTGGAGGAGTTGGAGG |  |
| **EBF1 primers** | | |
| 1 | CCATCTAACTCTGCGTCTTTGG | 134 |
|  | CTGGCCCTGTCTGTCGTAGA |  |
| 2 | CTCCATAGCCGCTGTTGTGA | 170 |
|  | ATGCATTCCTAACCCTCGCC |  |
| **EP300 primers** | | |
| 1 | CACAACAGGGCATATTTGGGC | 153 |
|  | TGGACAATACGCTCTGATACAGC |  |
| **EZH2 primers** | | |
| 1a | CTATTGCTGGCACCATCTGA | 191 |
|  | TCCAATCAAACCCACAGACTT |  |
| 1b | TTTATCAAAGATCCTGTGC | 100 |
|  | GTTAGTATATACAATGCCACCT |  |
| **FOXO1 primers** | | |
| 1 | TGTGCGGGGACTTCCAGG | 191 |
|  | GATGAGGTCGGCGTAGGAC |  |
| **GNA13 primers** | | |
| 1 | GATGGCGGACTTCCTGC | 192 |
|  | AAGGTGGACTTGCCGCT |  |
| 2 | TGGTGAAGATCCTGCTGCT | 186 |
|  | AGCCCCCCTGCCCTTAA |  |
| 3 | ACAGTGTGACATCAATACTTTTCCT | 189 |
|  | GCACCTTCTCCTCAAGCAAG |  |
| **GPR183 primers** | | |
| 1 | CGCTTTGCCTACACGAATAGC | 128 |
|  | GGCAGGTCATAAAGTTCACACC |  |
| **KMT2D primers** | | |
| 1 | TCGGGTAGGTTGGGTGC | 182 |
|  | GGTCCTCTTCTACGTAAGGAGCA |  |
| 2a | GGCACCTTCTCCTCCAAGTC | 199 |
|  | TGGAGACTGGGAGCTGGA |  |
| 2b | GGCACCTTCTCCTCCAAGTC | 105 |
|  | TCAGGGGGCGGAGGTT |  |
| 3 | CTGGTCCAGCAGCTGTCC | 191 |
|  | CCCATAAGGCCCTGACCCT |  |
| 4 | ACCCTCACCTGACTTTTCTTCT | 170 |
|  | AGGCAACCTGTACCCCA |  |
| 5 | CTGAATCAGCCCGACCCAAG | 188 |
|  | CTTCCCGCTCATCCTCCTG |  |
| 6 | TTGCCTGACATCCCTGAC | 189 |
|  | GGAGGAGGAGCTGCTT |  |
| **MFHAS1 primers** | | |
| 1 | ACACCCTGTCCATTGCTAG | 124 |
|  | ACGGTGTAGTGCAGTCCA |  |
| **MS4A1 primers** | | |
| 1 | CTCTTCCACATTGCCCTGGG | 143 |
|  | TTTGTCTGCACCATTTCCCAAA |  |
| **NFKBIZ primers** | | |
| 1 | ATGACCAGCCCGCTCAAC | 149 |
|  | GACGAGGCAGAGGAGAAGTC |  |
| **P2RY8 primers** | | |
| 1 | CTGCTGTTCCTCATCCCGTT | 146 |
|  | GTGACAAAGGCCAGCAAGAC |  |
| **PIK3CD primers** | | |
| 1 | GAGTGTGAGGGTCCCAGAGA | 154 |
|  | CTCGGGCAGGCAGATGAG |  |
| **SOCS1 primers** | | |
| 1 | CAGTCTCCACAGCAGCAGAG | 170 |
|  | CGTGATGCGCCGGTAATC |  |
| 2 | GCCTGCGGATTCTACTG | 193 |
|  | TGCCATCCAGGTGAAAG |  |
| **STAT6 primers** | | |
| 1 | CCCTGTCCTCACCCTCTTCA | 171 |
|  | TGCCCATGTTAGAACCCACC |  |
| **TNFRSF14 primers** | | |
| 1a | CTCACAGACAAGCAGTCCCTA | 215 |
|  | CTCCCCAGGGCACAGA |  |
| 1b | GGACACCCTGTGTCAGAACT | 120 |
|  | CTCCCCAGGGCACAGA |  |
| **TP53 primers** | | |
| 1 | TGGATGATTTGATGCTGTCCC | 127 |
|  | GGTGTAGGAGCTGCTGGTG |  |
| **ZFP36L1 primers** | | |
| 1 | GATTCTCTCTCGGACCAG | 139 |
|  | CTACCCTGGCTTAGTCATCT |  |
